# Retraction Note: Drying of a plasmid containing formulation: chitosan as a protecting agent

**DOI:** 10.1186/s40199-016-0150-0

**Published:** 2016-04-27

**Authors:** Nasir Mohajel, Abdolhossein R. Najafabadi, Kayhan Azadmanesh, Alireza Vatanara, Mohsen Amini, Esmail Moazeni, Amirabbas Rahimi, Kambiz Gilani

**Affiliations:** Aerosol Research Laboratory, Department of Pharmaceutics, School of Pharmacy, Tehran University of Medical Sciences, Tehran, Iran; Department of Pharmaceutics, Faculty of Pharmacy, Shahid Sadoughi University of Medical Sciences, Yazd, Iran; Department of Virology, Pasteur Institute of Iran, Tehran, Iran; Department of Medicinal Chemistry, School of Pharmacy, Tehran University of Medical Science, Tehran, Iran; Department of Nanobiotechnology, Pasteur Institute of Iran, Tehran, Iran

This article [1] has been retracted by the publisher because it was republished in the journal [2] due to an error during the process of journal transfer between publishers. BioMed Central apologizes to the authors and to readers for this error and for any inconvenience caused.
